# Ultrasound assessment of diaphragmatic dysfunction in non-critically ill patients: relevant indicators and update

**DOI:** 10.3389/fmed.2024.1389040

**Published:** 2024-06-18

**Authors:** Xin-Yu Yao, Hong-Mei Li, Bo-Wen Sun, Ying-Ying Zhang, Jian-Guo Feng, Jing Jia, Li Liu

**Affiliations:** ^1^Department of Anesthesiology, The Affiliated Hospital of Southwest Medical University, Luzhou, China; ^2^Anesthesiology and Critical Care Medicine Key Laboratory of Luzhou, Southwest Medical University, Luzhou, China; ^3^Department of Anesthesiology, Chengdu Fifth People’s Hospital, Chengdu, China

**Keywords:** diaphragm, diaphragm dysfunction, ultrasound, ultrasound indicators, ultrasound application, perioperative period, non-critically ill patients

## Abstract

Diaphragm dysfunction (DD) can be classified as mild, resulting in diaphragmatic weakness, or severe, resulting in diaphragmatic paralysis. Various factors such as prolonged mechanical ventilation, surgical trauma, and inflammation can cause diaphragmatic injury, leading to negative outcomes for patients, including extended bed rest and increased risk of pulmonary complications. Therefore, it is crucial to protect and monitor diaphragmatic function. Impaired diaphragmatic function directly impacts ventilation, as the diaphragm is the primary muscle involved in inhalation. Even unilateral DD can cause ventilation abnormalities, which in turn lead to impaired gas exchange, this makes weaning from mechanical ventilation challenging and contributes to a higher incidence of ventilator-induced diaphragm dysfunction and prolonged ICU stays. However, there is insufficient research on DD in non-ICU patients, and DD can occur in all phases of the perioperative period. Furthermore, the current literature lacks standardized ultrasound indicators and diagnostic criteria for assessing diaphragmatic dysfunction. As a result, the full potential of diaphragmatic ultrasound parameters in quickly and accurately assessing diaphragmatic function and guiding diagnostic and therapeutic decisions has not been realized.

## Introduction

**Figure 1:**
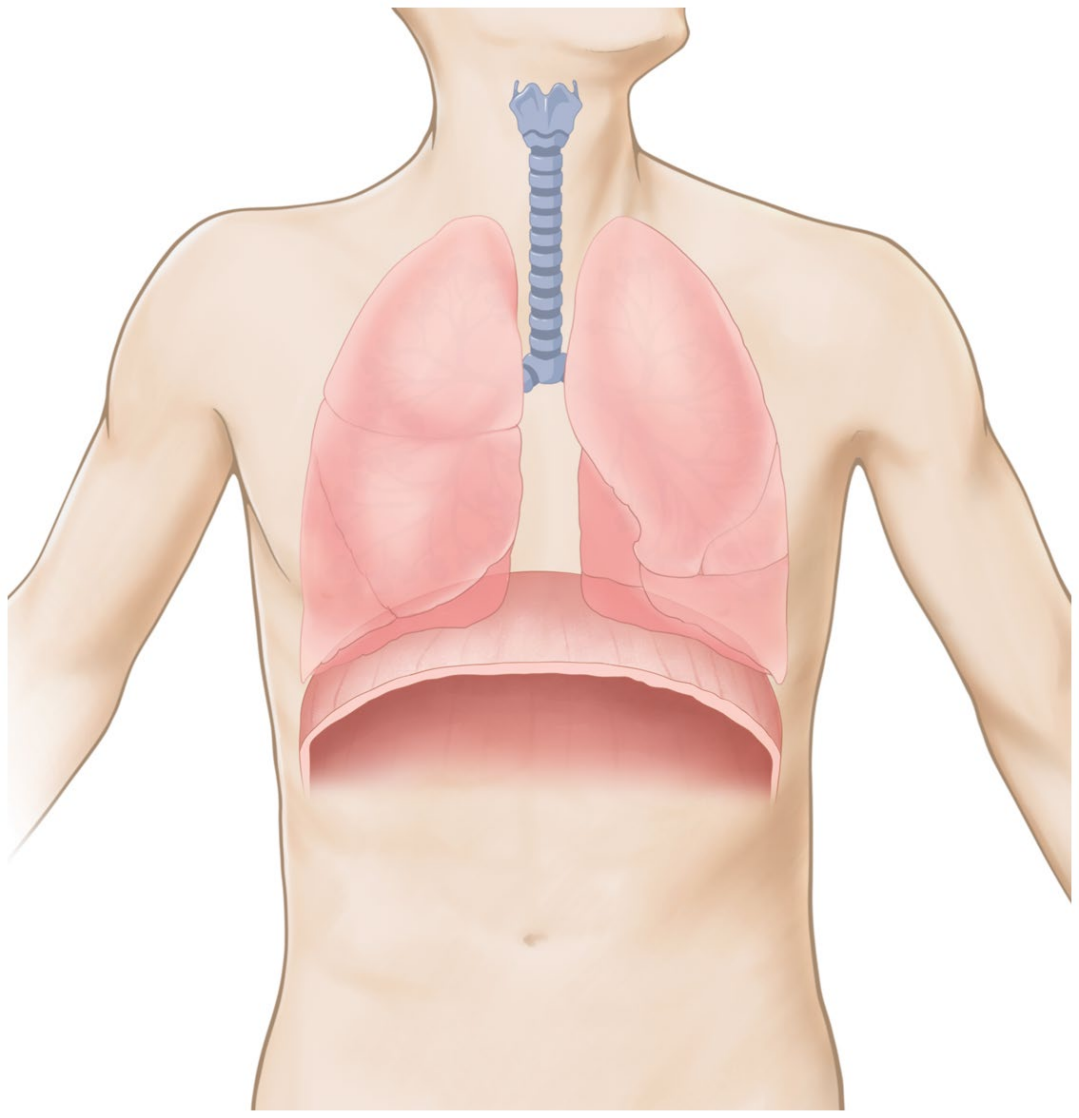
Location and morphology of the diaphragm.

The diaphragm is a thin, dome-shaped muscle that separates the thoracic and abdominal cavities (Figure 1). In a healthy adult, it is only 2–3 millimeters thick. Despite its small size, it is responsible for 60–80% of ventilation needs (1, 2). The diaphragm plays a crucial role in the respiratory muscle pump, aiding in coughing and the expulsion of secretions (3). It also reduces the risk of lung infections. Both mechanical ventilation and damage to the phrenic nerve can lead to diaphragmatic dysfunction (DD), characterized by an imbalance between the diaphragm’s ability to provide enough negative pressure for vital capacity and the workload imposed upon it. DD during mechanical ventilation (MV) is recognized as an important factor influencing clinical outcomes (4–9), and prolonged mechanical ventilation can result in decreased diaphragm thickness.

Studies have shown that for every 10% reduction in diaphragm thickness (DT) in critically ill patients, intensive care unit (ICU) mortality and hospitalization rates increase by 1.55- and 1.66-fold, respectively (10–12). More than 10 million patients worldwide require MV therapy each year, with approximately 30% of these patients needing extended ventilator use (13). Wasting atrophy of the diaphragm is a contributing factor to respiratory failure, and it is important to note that clinical symptoms may not occur until one’s diaphragmatic strength has decreased to 30% of its capacity (14). Early detection of DD is crucial, as early intervention can improve symptoms (15–17). However, there has been a gradual increase in the number of studies on non-critical patients in recent years, this suggests that diaphragmatic dysfunction is also common among perioperative non-critical patients. Therefore, the objective of this review was to analyze and summarize the indicators and criteria for ultrasound assessment of DD in non-critical patients. To achieve this, databases such as PubMed and Web of Science were searched with the aim of providing a reliable basis for clinical use.

Currently, there is no uniformity in the selection of ultrasound indicators and thresholds for diaphragmatic dysfunction, although there is an international expert consensus that a diaphragmatic excursion (DE) of less than 2 cm is the criterion for diagnosing diaphragmatic dysfunction (18), no article has been found to use this criterion. As a result, the main problem faced by clinicians is the lack of standardized criteria, while ultrasound is the preferred diagnostic tool for diaphragm dysfunction, a wide range of indicators and thresholds are summarized in the literature, which significantly affects clinicians’ judgment and delays early intervention.

## Ultrasound evaluation of diaphragmatic dysfunction

The gold standard for the diagnosis of diaphragmatic dysfunction is phrenic nerve stimulation and transdiaphragmatic pressure assessment (7, 19), however, these methods are invasive and not clinically applicable (20). In recent years, ultrasound has become a widely-used noninvasive technology (21), it allows noninvasive, reproducible, and safe assessment of the diaphragm’s anatomy and function (22–26). Two commonly used ultrasound modes are B-Mode and M-Mode (27, 28), and the key indicators of diaphragm function assessed by ultrasound include DE, diaphragm thickening fraction (DTF), and DT.

**Figure 2:**
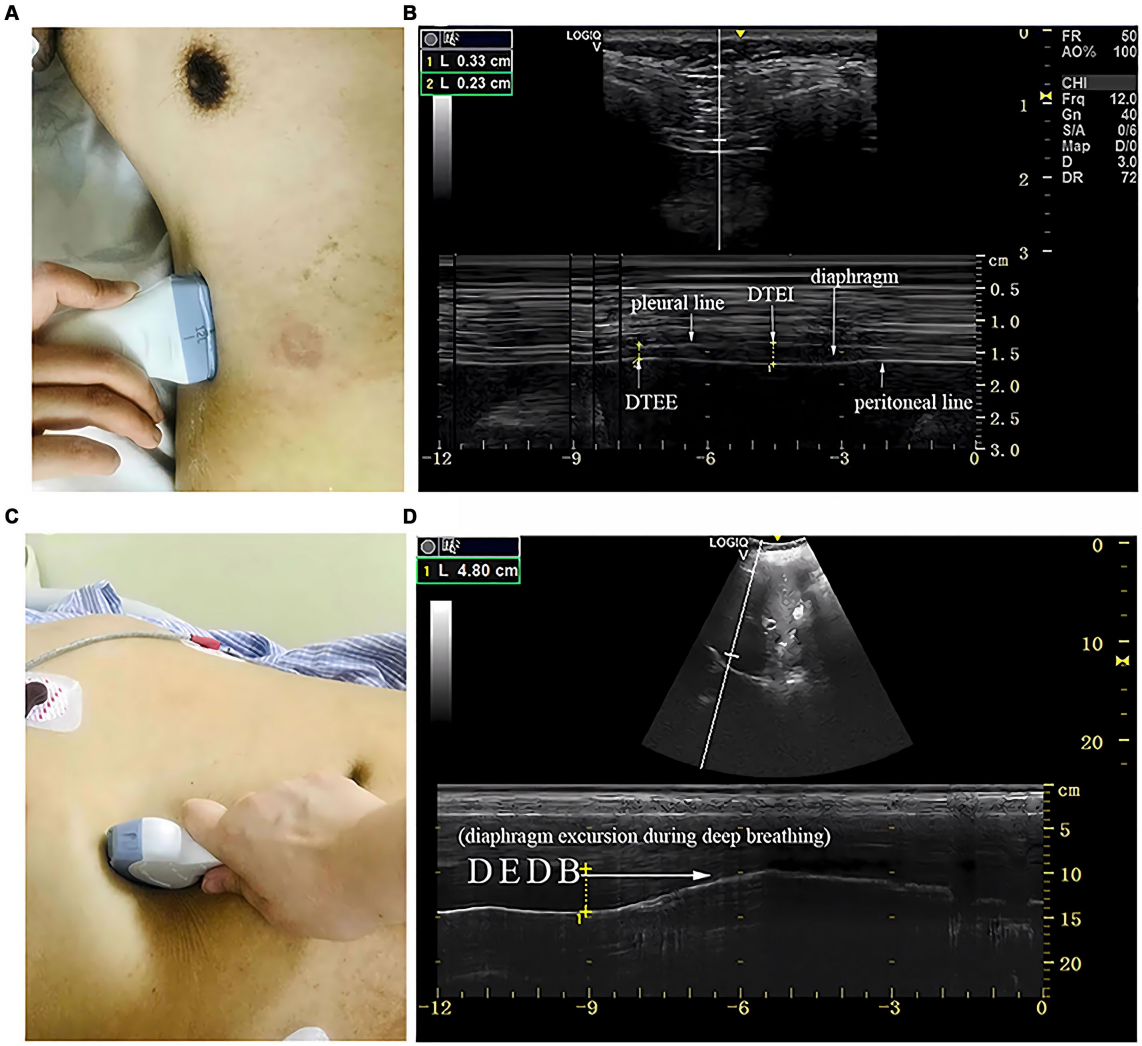
The measurement of diaphragmatic thickness and excursion. **(A)** A 10–15 MHz probe was placed at the zone of apposition. **(B)** The non-echogenic layer between the yellow markers indicates the thickness of the diaphragm at the end of expiration and inspiration. **(C)** A 2–5 MHz curved-array probe was placed under the costal margin. **(D)** The bright line indicates diaphragmatic excursion during deep breathing. DTEE, diaphragm thickness at end-expiratory; DTEI, diaphragm thickness at end-inspiration; DEDB, diaphragmatic excursion during deep breathing (30).

**Figure 3:**
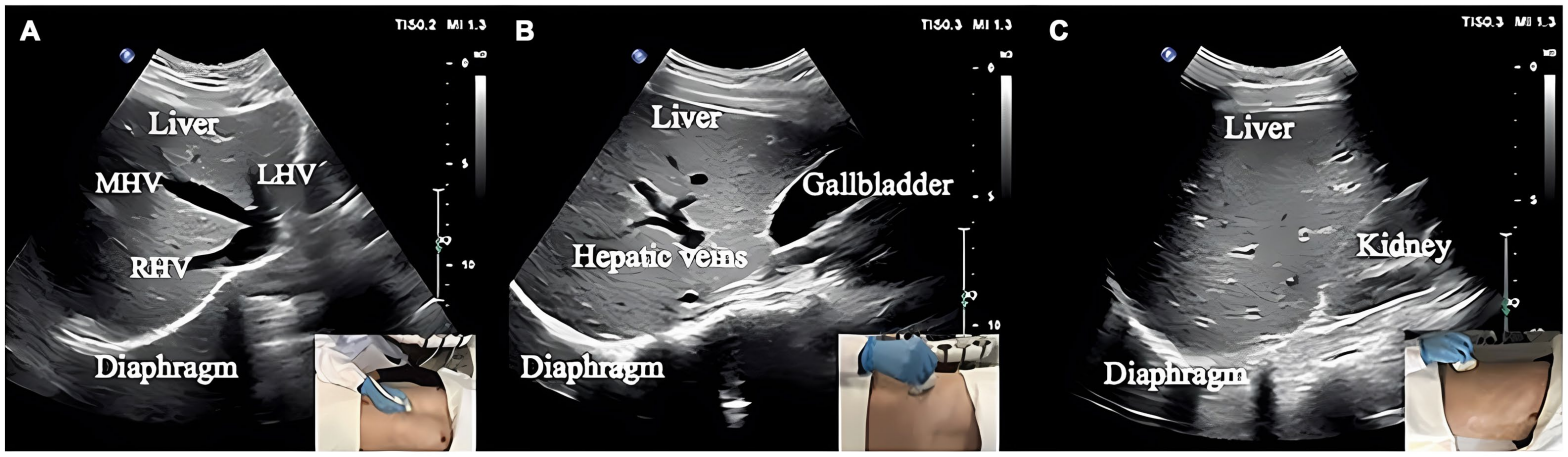
**(A)** Section I: Oblique section of the lower right costal arch through the second hepatic portal with the left hepatic vein (LHV), middle hepatic vein (MHV), and right hepatic vein (RHV) as anatomical markers. **(B)** Section II: Oblique section of the right intercostal passage through the first hepatic portal with the inferior vena cava, hepatic vein, and gallbladder as anatomical markers. **(C)** Section III: Sagittal section of the liver and right kidney with the right kidney and hepatorenal space as anatomical markers (31).

**Figure 4:**
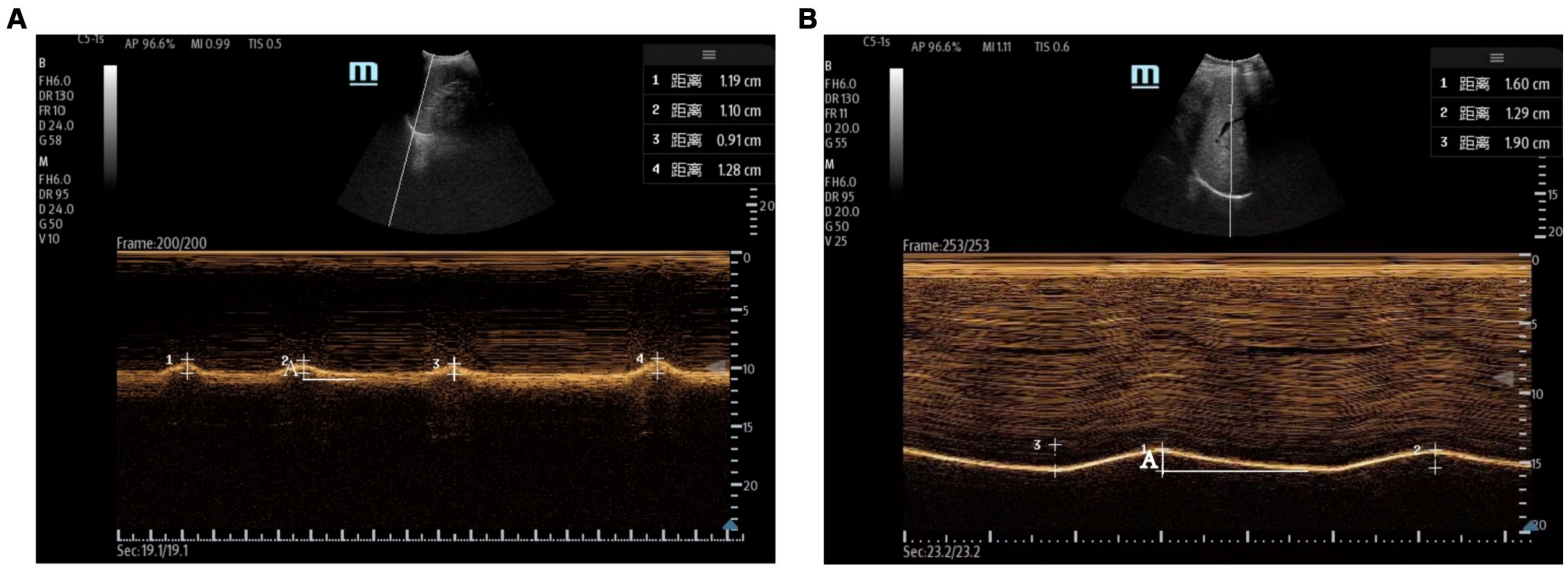
Evaluation of the diaphragm using ultrasound. **(A)** Left measurement of diaphragmatic excursion measured with the spleen as the acoustic window. **(B)** Right diaphragmatic excursion measured using the liver as an acoustic window. The total length of A is the diaphragmatic excursion for one respiratory cycle.

Measurements of DT and DTF require the use of a high-frequency linear ultrasound transducer (3–12 MHz). The patient should be in a semi-recumbent position, and the probe should be placed in the midaxillary line at ribs 8–10, perpendicular to the intercostal space. In B-Mode, the diaphragm can be visualized as a three-layered structure, with the upper hyperechoic layer being the pleura, the lower layer being the peritoneum, and the middle layer being the diaphragm (29) (Figures 2A,B). In contrast, DE measurements are performed using a low-frequency abdominal convex probe (3–5 MHz), the patient should be positioned at a 45-degree semi-recumbent angle, and the ultrasound probe should be placed parallel to the right costal margin at the right midclavicular line, using the transverse section of the liver as an acoustic window. Alternatively, the probe can be placed perpendicular to the costal margin to obtain a longitudinal section of the liver (Figures 2C,D). It is also possible to obtain diaphragm images at different interfaces using liver vessels as markers, however, this method is not commonly used (Figure 3). In B-Mode, the high echo shadow covering the liver surface represents the diaphragm, switching to M-Mode allows for the observation of the diaphragm waveform synchronized with the respiratory cycle (Figure 4B). On the left side, the probe is placed at the 8–10th rib along the midaxillary line, parallel to the intercostal spaces, the other methods are the same as for the right side (Figure 4A) (29). Ultimately, ultrasound is clinically reproducible (32) and has become an essential tool for most clinicians, its overall measurement failure rate has decreased from 27% a decade ago to 0.7% today, demonstrating the effectiveness of ultrasound technology (33).

Table 1 provides a comparison of the materials and methods used for assessing diaphragmatic dysfunction across the literature. Diaphragmatic ultrasound is widely used to assess diaphragmatic dysfunction in various medical conditions, including neuromuscular diseases (46, 47), chronic respiratory diseases, and conditions requiring intensive care (18, 21, 48), it helps clinicians diagnose and monitor conditions such as diaphragmatic paralysis or weakness. Additionally, diaphragmatic dysfunction can significantly impact weaning outcomes, thus, ultrasound can provide essential insights to predict the success of extubation (40, 49, 50). Ultrasound can also be used to counsel patients with respiratory failure in making decisions about the necessity and potential success of noninvasive ventilation (51). Additionally, it plays a crucial role in enhancing the understanding of ventilator management among patients with coronavirus 2019 disease (52).

**Table 1:** US assessment of diaphragmatic dysfunction in the literature.

	Cut-off values	Probe	Patient position	Measurement position	References
Diaphragm excursion (DE)	DE <10 mm	3.5–5-MHz convex ultrasound probe	Resting tidal breathing patients lying in the semirecumbent position	Right: placed between the midclavicular and anterior axillary lines Left: between the anterior and midaxillary lines	(34)
	Excursion <10 mm or negative	3.5-MHz US probe	Patients in the supine position	Right: right anterior axillary line Left: left midaxillary line	(9)
	DE <10 mm	3.5-MHz transducer	Semirecumbent position at 45°	NA	(35)
	DE <20 mm	2–5 MHz	NA	Probe aligned to the top of the diaphragm; placement not agreed upon	(18)
	Right: 2D-Mode < 10 mm or M-Mode < 11 mm Left: M-Mode < 11 mm	3.5-MHz US probe	The patient was supine with 30° head-of-bed elevation	Between the eighth and ninth ribs, on the midaxillary line	(36)
	DE <1 cm or diaphragmatic motion =0 cm	2.5–3.5-MHz phased-array transducer	Bedside with the patient in a semirecumbent position at 45°	Right: between the midclavicular and anterior axillary lines. Left: between the anterior and mid axillary lines	(37)
	DE <1 cm	2.5–3.5 MHz phased-array transducer	Bedside with the patient in a 45° semi-recumbent position	Midaxillary line	(38)
	Greater than 75% reduction in diaphragmatic excursion in the *VS* test	4-MHz curvilinear transducer	Patient in a semi-sitting position with the head elevated by approximately 30°	Right anterior axillary and midclavicular lines	(39)
	Men: DE <1 cm Women: DE <0.9 cm	2.5–3.5 MHz	Standing position	Right: between the midclavicular and anterior axillary lines. Left: between the anterior and mid axillary lines	(32)
	DE <1 cm	4-MHz linear probe	NA	NA	(40)
Diaphragm thickness (DT) Diaphragm thickness fraction (DTF)	DTF <30%	Linear probe (6–13 MHz)	Semi-recumbent position (head raised by 40°)	Right midaxillary line	(41)
	Thickness reduction >10% from baseline	7–12 MHz	NA	Midaxillary line or slightly ventral, approximately between the 8–11th ribs	(18)
	DTF <20%	7.5–10 MHz probe	Semi-recumbent position	Between the 8–10th intercostal ribs	(42)
	DT-exp <2 mm	9.0-MHz probe	NA	In line with the right intercostal space between the anteroaxillary and midaxillary lines	(43)
	DT <20%	7.5–10.0-MHz transducer	Standing position	The eight and nine intercostal spaces in the right midaxillary line	(44)
	DTF < 30%	10-MHz linear probe	NA	NA	(40)
	DTF <20%	Linear-array probe (5–10 MHz)	NA	In the right 8th or 9th intercostal space	(45)

DTF, Diaphragm thickness fraction = (inspiratory thickness − expiratory thickness)/ expiratory thickness × 100; DE, diaphragm excursion; DT-exp, Diaphragmic thickness during static inspiratory or expiratory phases, corresponding to functional residual air volume; NA, Not mentioned in the literature; VS, voluntary sniff.

In anesthesiology, diaphragm ultrasound helps determine the residual muscle relaxation in patients under general anesthesia, addressing the complexities and interferences associated with the gold standard train-of-four ratio procedure (53), it is possible to predict and prevent postoperative pulmonary complications in surgical patients, including those undergoing heart surgery, thoracic surgery, and upper abdominal surgery (34, 54–58). In rehabilitation medicine, diaphragm thickness is positively correlated with patients’ functional scores and functional independence scores before and after rehabilitation, suggesting that diaphragm thickness can influence patients’ rehabilitation progress (59). Overall, diaphragm ultrasound has become a valuable tool in routine clinical practice, particularly for assessing diaphragmatic function in various medical conditions.

## Perioperative ultrasound assessment of diaphragmatic dysfunction

### Preoperative assessment of DD

Ultrasound is often used in clinical trials in patients undergoing elective surgery, normal healthy people, and critically ill patients (32, 48, 55). Notably, patients with bilateral DD experience a significant 75% reduction in forced vital capacity (FVC) (60), it can also lead to impaired lung ventilation, resulting in pneumonia or atelectasis (61–63). Therefore, preoperative DD not only affects a patient’s quality of life but also impacts their prognosis and survival after surgery. Huh et al. demonstrated a significant association between preoperative DD and prolonged postoperative mechanical ventilation in lung transplantation patients using a DE measurement of <1 cm (odds ratio [OR]: 2.79, *p* < 0.05). Additionally, patients with preoperative DD had a 15% probability of developing persistent DD 1 year after surgery (64).

Tension pneumoperitoneum is a known cause of DD, and a case report series assisted surgeons in identifying the cause of postoperative respiratory failure in patients by using an ultrasound measurement of diaphragm thickness ratio(diaphragm thickness of maximal inspiration/that of end-expiration) < 1.2 (65), despite diaphragm thickness ratio normalization after 10 days, delayed diagnosis negatively impacts the patient’s prognosis, and DD in patients undergoing cardiac surgery is one of the most overlooked complications (66). A maximum preoperative DTF <38.1% is associated with pulmonary complications after cardiac surgery (OR: 4.29 *p* = 0.02) (54), and preoperative respiratory muscle training reduces the incidence of postoperative pulmonary complications by 50% (67), this reduction occurs because the clinical presentation of DD varies from asymptomatic in mild cases to requiring prolonged mechanical ventilation or even death in severe cases (68, 69). Thus, ultrasonography can be used to detect abnormal diaphragmatic function early and prompt clinicians to intervene. Overall, there is a lack of studies on preoperative DD, nevertheless, the relevance of assessing diaphragmatic function preoperatively for postoperative prognosis is worth exploring.

Preoperative DD not only has negative effects on early clinical outcomes but also jeopardizes long-term lung function, resulting in negative outcomes such as reduced total lung volume and functional capacity, even in patients who undergo successful surgery. Therefore, patients diagnosed with DD preoperatively require individualized surgical plans and intraoperative management from surgeons and anesthesiologists, this may include preoperative monitoring of respiratory function, X-rays, and exertion spirometry assessment (70–72), these preparations can help reduce postoperative complications in patients.

### Intraoperative assessment of DD

Diagnosis of intraoperative DD by ultrasound is challenging due to the position and location of surgery and the requirement for muscle relaxants in patients under general anesthesia. As a result, intraoperative studies have primarily focused on patients undergoing shoulder surgery who have received brachial plexus blocks, these blocks involve sensory numbing of the fourth and fifth cervical nerves, while Cervical 3 to Cervical 5 (C3–C5) MV blocks can cause varying degrees of diaphragmatic paralysis (73, 74). Consequently, the most common complication of brachial plexus blocks is ipsilateral diaphragmatic paralysis from phrenic nerve blocks. The reported incidence of ipsilateral hemidiaphragmatic paralysis after supraclavicular brachial plexus nerve block ranges from 67 to 80% (75, 76), with a much higher incidence when using the interosseous groove approach, especially if a high volume injection of 20 mL is used (74). This higher incidence could be attributed to the closer proximity of the block site to the cervical plexus, while reducing the concentration or dose of local anesthetic can lower the incidence of diaphragmatic paralysis, it comes at the expense of diminished analgesia (77). Therefore, it is important to explore how to find a critical value that achieves satisfactory pain relief while avoiding diaphragmatic paralysis. Notably, a decrease in DE of more than 75% indicates complete paralysis of the diaphragm (39), thus, utilizing ultrasound to assess diaphragmatic function in patients undergoing shoulder surgery with brachial plexus nerve block allows for early detection of the risk of diaphragmatic paralysis, which in turn enables appropriate measures to be taken, such as endotracheal intubation under general anesthesia, to prevent intraoperative respiratory distress.

### Postoperative assessment of DD

In 1993, Fratacci et al. demonstrated that thoracotomy and lobectomy severely affect the active contraction of the diaphragm, leading to diaphragmatic depression (78), diaphragmatic contraction was markedly attenuated only 2 h after thoracic surgery (79). However, due to the limitations of the conditions at the time, only rough conclusions could be drawn. The definition of DD was further refined by the introduction of ultrasound in 2010, it was reported that operatively measured DE was significantly reduced compared with nonoperative measurements, highlighting the need to use ultrasound in the perioperative period to avoid errors in whole-body pulmonary function assessments (80). In an experiment of continuous diaphragm ultrasound assessment in 107 mechanically ventilated patients, 47 (44%) patients had a decrease in diaphragm thickness of more than 10%, 13 (12%) had an increase in diaphragm thickness of more than 10%, and 47 (44%) had no change in diaphragm thickness during the first week of mechanical ventilation (81). Although there was no significant difference in the results among these three groups, this study showed that ultrasonography could be used to monitor changes in the diaphragm during mechanical ventilation. Moreover, the indisputable value of dynamic diaphragm assessment through ultrasound has been further highlighted by subsequent studies.

Spadaro et al. demonstrated that the incidence of DD was higher in patients undergoing both video-assisted thoracoscopic surgery (VATS) and open thoracic surgery at a DE <10 mm (34); however, the incidence of VATS was slightly lower than that of open thoracic surgery, which is in accordance with the less invasive and better recovery characteristics of VATS. Importantly, this study demonstrated a correlation between postoperative pulmonary complications and DD at 24 h postoperatively (OR: 5.5, *p* = 0.002). In contrast, Daniel et al. compensated for Spadaro et al.’s shorter monitoring duration by using ultrasound to measure DE and DTF in patients undergoing thoracic surgery, their study included preoperative, post-extubation, and three-day postoperative assessments. They observed a significant decrease in DE following surgical interventions, both post-extubation and at the three-day mark after surgery, at 3 days, there was also a significant decrease in DTF following a similar time course. However, DE was easier to assess and more reproducible than DTF, making it more suitable for perioperative assessment of diaphragmatic dysfunction. Additionally, persistent diaphragmatic dysfunction was associated with an increased risk of pulmonary infections (OR: 9.0, *p* = 0.001), this finding is consistent with the results obtained by Spadaro et al. (82), who demonstrated that 68% of patients experienced immediate post-extubation diaphragmatic dysfunction. Spadaro et al. also reported an incidence of 68% (34) for diaphragmatic dysfunction at 3 days postoperatively, which suggests that diaphragmatic dysfunction is self-recovering but takes some time. Furthermore, the occurrence of postoperative diaphragmatic dysfunction lasting at least 3 days is related to the duration of hospitalization. However, the exact duration of postoperative diaphragmatic dysfunction has not yet been definitively determined.

In a study of cardiac surgery, Tralhão et al. extended the monitoring time to the fifth postoperative day and found that DE and DTF decreased on the first postoperative day but returned to preoperative levels by the fifth day (38). This suggests that diaphragm dysfunction occurs at a high incidence in cardiothoracic surgery. Additionally, postoperative diaphragmatic dysfunction in patients undergoing lobectomy leads to a decrease in static balance, thereby affecting the patient’s daily life (83). Nevertheless, ultrasound can be used to continuously monitor diaphragm function and dynamically observe diaphragm recovery in the postoperative period, this can improve the predictive value of adverse outcomes in postoperative patients.

The incidence of DD after cardiac surgery is as high as 38% (56, 66), and persistent DD occurs in 8% of patients (57). When it occurs, it can lead to serious complications, and the incidence of postoperative DD in patients with congenital heart disease is 6.3% (84). Moury et al. found a 20% probability of a 20% reduction in the thickness of DTF at the 75% probability threshold by employing continuous ultrasonographic monitoring of DTF at pre-, mid-, and post-spontaneous breathing trial (SBT) time points (42). Despite the association between thickness reduction and prolonged hospital stay, the authors did not investigate to what extent such reductions constitute DD. Given the high incidence of thickness reduction in the postoperative period, further exploration of this question is warranted.

When DE <1 cm was used as a diagnostic criterion for DD, the incidence of bilateral DD and unilateral DD persisting until 3 days postoperatively was 36 and 12%, respectively (38). In a study by Laghlam et al., persistent DD after cardiac surgery was investigated (57), DD was defined as DE <9 mm in women and DE <10 mm in men for calm breathing, and DE <16 mm in women and DE <18 mm in men during sniffing breaths, the incidence of DD remaining on postoperative day 7 in the presence of spontaneous breathing was found to be 8% (10/122). Although there was a decrease in DE compared to patients without DD, no preoperative risk factors were identified for persistent postoperative DD.

Persistent DD can severely impair respiratory function in the postoperative period, leading to an increased frequency of pneumonia and reintubation. However, it is noteworthy that Tralhão et al. (38) reported a different trajectory, with DE returning to preoperative levels within 5 days postoperatively. Possible explanations for this discrepancy include the small sample size and relatively low age of the population studied by Tralhão et al., as well as the absence of prevalent neocoronary pneumonia at the time of their study. In addition, Pasero et al. found that 21 and 25% of patients had persistent diaphragmatic dysfunction on the right and left sides using a threshold of DTF <30% (85). Meanwhile, the incidence at DSBT was as high as 38% when DTF <20% was utilized as the threshold (66). This discrepancy is significant compared to the 75% incidence reported by Moury et al. Several factors may explain this difference: (1) patients in the study by Moury et al. had a longer extracorporeal circulation time, which is strongly correlated with diaphragmatic dysfunction; (2) the prevalence of preoperative DD was 11% in their study, which is higher than the 7% reported by Pasero et al.; and (3) there were inconsistencies in the criteria used for diagnosing DD. Therefore, despite the relatively low incidence of persistent DD after cardiac surgery, it still has a significant impact on patient prognosis and necessitates the use of ultrasound-assisted monitoring.

Diaphragmatic ultrasound was described for the first time in the context of the recovery period after cardiac surgery. It was determined that ultrasound can be utilized as part of clinical practice for initial postoperative rehabilitation and follow-up assessment (86). While diagnostic criteria such as DE, TF, and DTF have yielded inconsistent clinical outcomes in assessing DD, DE is the preferred index for most investigators due to its high reproducibility and accuracy. Nevertheless, there is still a lack of large sample size multicenter studies to further validate the clinical applicability of each index.

Postoperative diaphragmatic dysfunction was diagnosed according to an ultrasound diaphragm thickness ratio < 1.2 in a patient who underwent gastrointestinal endoscopic surgery (65), this is a rare occurrence and suggests that flatulence after abdominal hyperextension is one of the causes of DD. Notably, cholecystectomy leads to diaphragmatic damage (87); however, this damage typically resolves within 24 h (88). However, Benhamou et al. concluded that abdominal pneumoperitoneum does not impair diaphragm function after laparoscopy (89). Nevertheless, there remains a dearth of reports elucidating the threshold for DD induced by laparoscopy and the timeframe course for the diaphragm for diaphragmatic recovery to baseline. Given the widespread adoption of laparoscopy for minimally invasive upper abdominal surgery, understanding these dynamics is crucial. Furthermore, postoperative DE decreases after upper abdominal surgery, leading to a shift from predominantly abdominal to predominantly ribcage respiration. This alteration can predispose postoperative patients to pulmonary complications such as atelectasis and hypoxemia (90). Hence, incorporating ultrasound evaluation into postoperative care protocols for upper abdominal surgery is imperative.

A study by Crothers et al. followed up lung transplant patients and found that the prevalence of diaphragmatic dysfunction decreased from 66 to 22% at 3 months postoperatively (91), this suggests that diaphragmatic dysfunction may still be present in the months following surgery; however, the prevalence decreases over time. Similar results have been observed in children (92). Ultimately, early evaluation and treatment of diaphragmatic dysfunction can improve patient prognoses.

## Ultrasound assessment of diaphragmatic dysfunction in nonsurgical patients

**Figure 5:**
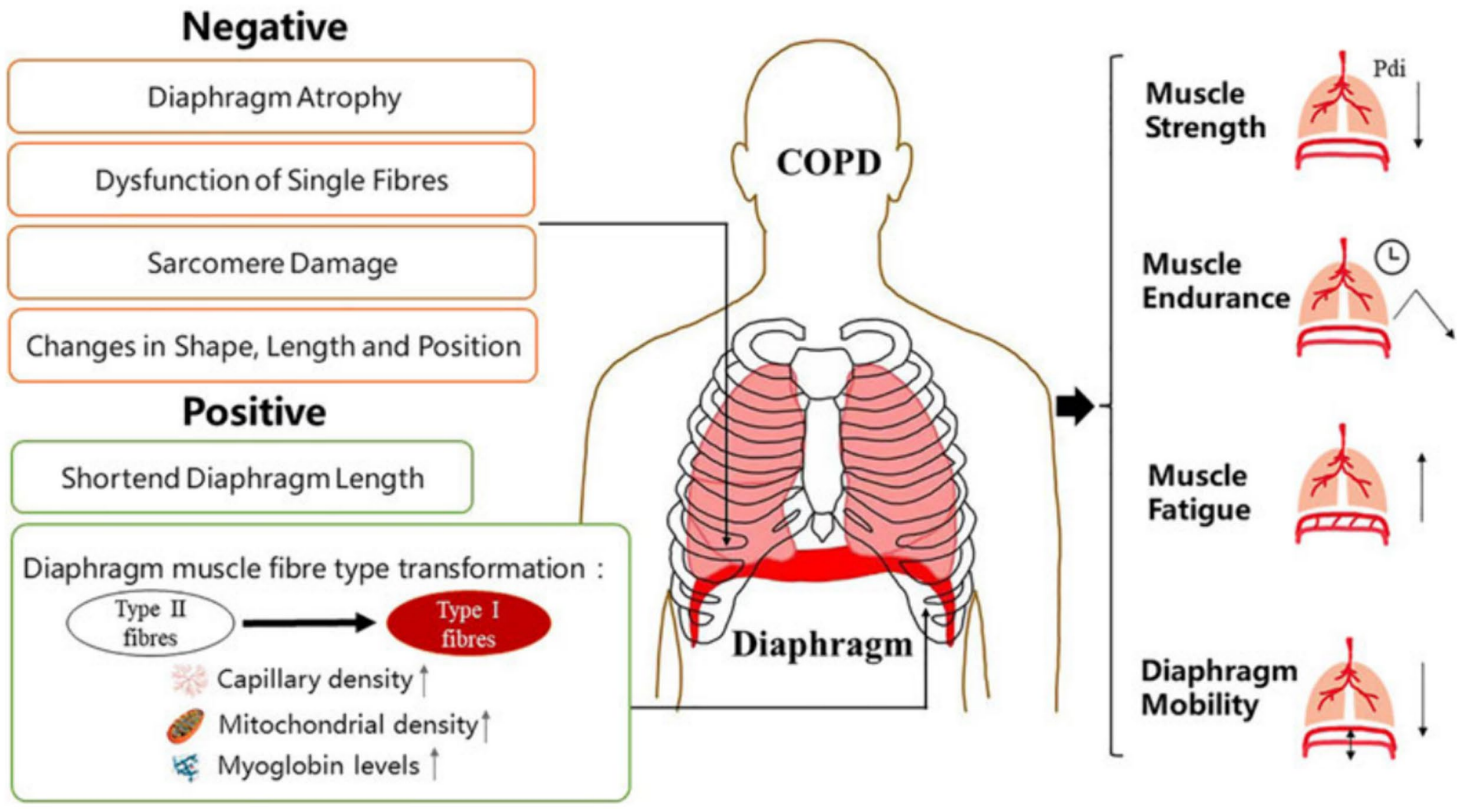
Manifestations of diaphragm dysfunction in COPD. Diaphragm dysfunction in patients with COPD is mainly manifested in structural and functional changes. Changes in diaphragm structure include both negative and positive changes. The function of diaphragm depends largely on its physiological characteristics at the structural level. COPD, chronic obstructive pulmonary disease (95).

There are few studies related to ultrasound in nonsurgical patients, such as outpatients with chronic obstructive pulmonary disease (COPD) and interstitial lung disease (ILD). However, evidence suggests that ultrasound monitoring of diaphragm function is useful in assessing a range of lung diseases (48, 93). In patients with COPD, lung hyperinflation causes the diaphragm to shift caudally, negatively affecting its function (94). The clinical presentation of COPD patients is shown in Figure 5. In the past, the assessment of patients with COPD mainly involved using the 6-min walking test and FEV1/FVC evaluation (forced expiratory volume in the first second/forced vital capacity). In recent years, ultrasound has also played an important role in analyzing patients with COPD, and DE is an essential indicator of decreased exercise tolerance and dyspnea, which are related to lung function and respiratory muscle strength (96, 97). The lower normal limit value of DE in healthy subjects is 3.3 cm for men and 3.2 cm for women during deep breathing (98). This corresponds to the fact that diaphragmatic mobility is greater in men than in women. However, the diagnostic threshold for DD is significantly higher than that used in most clinical trials.

The occurrence of DD in COPD leads to a significant decrease in DTF, TF, and DE (99). Notably, ultrasound monitoring of the diaphragm, both in outpatients and hospitalized COPD patients, can effectively assess the disease status of patients (100–105). Moreover, early detection of diaphragmatic dysfunction can help in formulating relevant strategies to reduce the occurrence of adverse clinical outcomes. However, the lack of uniformity in the ultrasound criteria for diagnosing DD will result in a much higher rate of leakage and misdiagnosis. Additionally, a large number of sample sizes and experiments are needed to further validate the accuracy of the diagnostic criteria.

Bernardinello et al. conducted TF ultrasound measurements on outpatients with ILD for several months, they found that a TF <30% was the diagnostic criterion for DD. Of the 82 ILD patients followed up, 24 experienced DD, resulting in an incidence rate of 29%. Furthermore, DD was more likely to occur in patients with connective tissue disease (CTD-ILD) than in healthy subjects. In their study, TF <30% was found to be an independent predictor of moderate/severe dyspnea (OR: 3.8, *p* = 0.009 and OR: 6.3, *p* = 0.021, respectively) (41). On the other hand, idiopathic pulmonary fibrosis (IPF) patients did not exhibit similar results, this led to the conclusion that CTD-ILD, a systemic disease that may decrease muscle strength, is different from IPF, a chronic lung disease in which muscle strength is better maintained. These findings are in line with previous research (106). Additionally, CTD-ILD patients who developed DD were more likely to experience severe dyspnea. Therefore, identifying risk factors for DD in CTD-ILD patients could help prevent poor clinical outcomes. Meanwhile, another study by Santana et al. demonstrated that DE correlates with ILD severity in ILD patients, they also found that FVC% <60 is highly accurate for predicting DD (107). In clinical practice, diaphragmatic ultrasound imaging has a high sensitivity and specificity for identifying reduced DE in ILD patients with FVC% <60. By combining ultrasound with lung function indices, it becomes easier to monitor ILD patients after surgery and can also serve as a prompt for physicians to reduce the use of medications, such as corticosteroids, that may lead to myopathy.

The assessment of diaphragmatic dysfunction using ultrasound in patients with neuromuscular diseases is a critical area of study due to the essential role the diaphragm plays in respiration. Neuromuscular diseases, such as amyotrophic lateral sclerosis (ALS), Duchenne muscular dystrophy (DMD), stroke, myasthenia gravis (108), and Lambert-Eaton syndrome (46), can lead to significant diaphragmatic weakness or paralysis, severely affecting respiratory function. Consequently, in cases of acute myasthenic crisis, patients may experience acute respiratory failure requiring invasive ventilation. Transitioning to ALS, early detection poses challenges, with low survival rates primarily attributed to respiratory muscle involvement. Timely intervention is critical, as diaphragmatic ultrasound can effectively predict FVC <50% by measuring parameters such as DE (<5.5 cm) during deep breathing. This comprehensive assessment facilitates prompt intervention for respiratory failure, potentially improving patient prognosis (109).

Stroke also affects respiratory function to some extent, resulting in a notable reduction in diaphragm mobility and lung function among affected patients (110). This diminished respiratory capacity can heighten the vulnerability of stroke patients to pulmonary infections. Similarly, patients with DMD exhibit lower DE and DTF compared to healthy adults (111, 112). Although there is no cure, diaphragmatic ultrasound can provide a clinical basis for assessing diaphragmatic function. In other words, ultrasound is the preferred tool for identifying patients who may have experienced diaphragmatic dysfunction before they display clinical symptoms, enabling early intervention.

## Discussion

Although ultrasound has become a commonly used tool for diagnosing diaphragmatic dysfunction in recent years due to its noninvasiveness and reusability, there is still confusion regarding the use of diagnostic indicators and criteria. Some literature suggests that diaphragmatic involvement is bilateral (66). In contrast, some studies have demonstrated that DD may be unilateral and associated with specific surgical procedures, such as lung resection (34). During quiet breathing in healthy individuals, the lower limit of normal DE is 0.9 cm and 1 cm in women and men, respectively. Meanwhile, during deep breathing, the lower limit of normal DE is 3.3 cm and 3.2 cm for women and men, respectively (98). While both lower values have been used in different articles to diagnose DD, in a randomized controlled study of patients treated with nerve blocks, DD was categorized into complete, partial, and no diaphragmatic dysfunction categories based on decreases in DE from baseline of >70%, 25–70, and < 25% (113), respectively. Overall, the metrics used to diagnose DD through ultrasound have also not been standardized and include DE (9, 34–40), DT, and DTF (18, 41–45).

By analyzing and summarizing the literature, this review found that DE is the most commonly used index. A DE measurement of <1 cm is often used to diagnose diaphragmatic dysfunction. In clinical practice, it has been observed that most normal patients have a DE between 1–2 cm, with a few exceeding 2 cm. These findings are consistent with the data obtained by Boussuges et al. regarding DE in normal subjects (32). However, there is an international consensus among experts on ultrasound diagnosis of diaphragmatic dysfunction in critically ill patients. According to this consensus, a DE <2 cm from baseline can be considered as the critical value for diagnosis of DD (18), this conclusion contradicts the findings of our review. However, it should be noted that this consensus is specifically for ICU patients, who often have additional conditions such as diaphragmatic edema, inflammation, and pulmonary atelectasis. These conditions require a greater DE to maintain normal tidal volume. Additionally, the thickness of the diaphragm is not standardized due to diaphragmatic edema and other factors, therefore, a DE <2 cm cannot be used as a diagnostic criterion for non-critical patients. In conclusion, diaphragmatic ultrasound plays an important role in clinical practice, but there is no consensus on the diagnostic criteria for non-critical patients. Currently, a DE <1 cm is the most reasonable criterion, but more clinical studies are needed in the future to confirm and supplement this criterion. Secondly, through literature review, we also found that the incidence of diaphragmatic dysfunction caused by brachial plexus block is very high. However, brachial plexus block has now become a common anesthesia method in orthopedic surgery, which can avoid the adverse effects of general anesthesia. In the future, further research can be conducted on another approach or concentration to reduce diaphragmatic dysfunction caused by brachial plexus block. Furthermore, the use of ultrasound at the bedside is limited by the presence of poor acoustic windows in some outpatients and ICU patients (93, 114, 115) and unfavorable imaging environments in obese patients (25).

In recent years, computed tomography (CT) has emerged as a new tool for characterizing diaphragmatic function, it enables visual assessment of diaphragm density, thickness, and height (116), facilitating the prediction of reintubation rates in patients in the ICU (117). Additionally, CT allows for the static assessment of the diaphragm and observation of morphological changes over time. Looking ahead, alongside ultrasound, CT is poised to become an indispensable tool for comprehensive assessment of diaphragm function.

## Conclusion

In clinical practice, ultrasound remains a commonly used tool for assessing DD, it is not only noninvasive but can also be performed at the bedside, ensuring good patient compliance. Perioperative ultrasound assessment of diaphragm function can help in preoperative preparation, intraoperative monitoring, and postoperative evaluation. It allows clinicians to promptly and accurately assess diaphragm function and guide subsequent treatment strategies. However, more clinical data are required in the future to complement and support this review, with the ultimate goal of reaching a consensus on ultrasound assessment in non-critical patients.

## Author contributions

X-YY: Writing – original draft. H-ML: Writing – review & editing. B-WS: Writing – review & editing. Y-YZ: Writing – review & editing, Investigation. J-GF: Writing – original draft, Resources. JJ: Writing – review & editing, Methodology. LL: Writing – review & editing, Conceptualization.
